# Population dynamics and antimicrobial resistance of *Salmonella* Derby ST40 from Shenzhen, China

**DOI:** 10.3389/fmicb.2022.1065672

**Published:** 2022-12-20

**Authors:** Miaomiao Luo, Yiying She, Yixiang Jiang, Li Xie, Chao Yang, Yaqun Qiu, Rui Cai, Yinghui Li, Liangcai Xu, Lulu Hu, Lei Wang, Shuang Wu, Qiongcheng Chen, Xiaolu Shi, Min Jiang, Qinghua Hu

**Affiliations:** ^1^School of Public Health, Shanxi Medical University, Taiyuan, China; ^2^Shenzhen Center for Disease Control and Prevention, Shenzhen, China; ^3^School of Public Health, University of South China, Hengyang, China; ^4^The Center for Microbes, Development and Health, CAS Key Laboratory of Molecular Virology and Immunology, Institute Pasteur of Shanghai, Chinese Academy of Sciences, Shanghai, China; ^5^Shenzhen Futian District Center for Disease Control and Prevention, Shenzhen, China

**Keywords:** *Salmonella* Derby, phylogenetic analysis, foodborne illness, virulence factor, whole-genome sequencing, antimicrobial resistance

## Abstract

*Salmonella enterica* subsp. *enterica* serovar Derby (*S.* Derby) is one of the most common serotypes responsible for salmonellosis in humans and animals. The two main sequence types (ST) observed in China are ST40 and ST71, with ST40 presently being the most common in Shenzhen. Recent years have seen an increasing number of cases of salmonella caused by ST40 *S*. Derby, but the epidemiology is not clear. We gathered 314 ST40 *S*. Derby isolates from food and patient samples for 11 years in Shenzhen; 76 globally prevalent representative strains were also collected. Whole-genome sequencing (WGS) combined with drug resistance phenotyping was used to examine population structural changes, inter-host associations, drug resistance characteristics, and the food-transmission risks of ST40 *S*. Derby in Shenzhen over this period. The *S. enterica* evolutionary tree is divided into five clades, and the strains isolated in Shenzhen were primarily concentrated in Clades 2, 4, and 5, and thus more closely related to strains from Asian (Thailand and Vietnam) than European countries. Our 11-year surveillance of *S*. Derby in Shenzhen showed that Clades 2, 4, and 5 are now the dominant epidemic branches, and branches 2 and 5 are heavily multi-drug resistant. The main resistance pattern is ampicillin-tetracycline-ciprofloxacin-chloramphenicol-nalidixic acid-streptomycin-sulfamethoxazole/trimethoprim. This may lead to a trend of increasing resistance to ST40 *S*. Derby in Shenzhen. Using a segmentation of ≤3 SNP among clone clusters, we discovered that Clades 2 and 4 contained multiple clonal clusters of both human- and food-derived strains. The food-derived strains were mainly isolated from pig liver, suggesting this food has a high risk of causing disease outbreaks in Shenzhen.

## Introduction

Non-typhoidal *Salmonella* (NTS) is a significant zoonotic food-borne pathogen and one of the most serious public health issues worldwide (Majowicz et al., 2010). NTS can result in a range of clinical presentations, most frequently appearing as very minor gastrointestinal symptoms; however, the infection can occasionally become life-threatening, especially in young children and older adult individuals (Ruiz et al., 2004). The global burden of NTS gastroenteritis is estimated to be 93.8 million cases and 155,000 deaths per year (Balasubramanian et al., 2019). Food of animal origin is a primary vector of human *Salmonella* infection and has been linked to outbreaks of human *Salmonella enterica* subsp. *enterica* serovar Derby (*S.* Derby), a serotype that primarily affects high-risk people and was first isolated by Peckham in 1923 from pork patties that caused food poisoning (Zheng et al., 2017). In Europe, *S.* Derby is the most common serotype isolated from pork. It accounts for 22.9% of all isolates, followed by *S*. 4,[5],12:i:- (i.e., the monophasic variant of *S. typhimurium*) (22.3%) and *S. typhimurium* (20.6%) (EFSA, 2016). In the United States, *S.* Derby is the fourth most common isolate of non-human origin (Nguyen et al., 2015), while in China, it is the most common isolate from the sera of slaughtered pigs and the third most common from sera of clinical cases (Deng et al., 2012). Hence, *S.* Derby is the most common *Salmonella* serotype found in many countries, including countries in Europe, America, and Asia, and has been linked to several food-borne disease outbreaks in recent years.

In 1946, *S.* Derby caused an epidemic in Australia that affected 68 infants and resulted in the deaths of 10 babies (Mushin, 1948). In the United States, an outbreak in 1963 associated with contaminated eggs involved 822 cases in 53 hospitals (Sanders et al., 1963). In Germany, an outbreak between late 2013 and early 2014 associated with pork contaminated with *S.* Derby involved 145 patients, the majority of whom were elderly (Simon et al., 2018). In addition to these well-known foodborne outbreaks, *S.* Derby is frequently implicated in causing widespread human cases not associated with any specific food. *S.* Derby is the fifth most common serovar isolated from humans in Europe and caused 612 confirmed cases in 2017. Cai et al. investigated the contamination of pork samples from slaughterhouses and farmers’ markets in Jiangsu Province, and *S.* Derby was the serovar most frequently isolated (Cai et al., 2016). In China, *S.* Derby is the third most commonly reported serovar in clinical cases (Ran et al., 2011) and the most frequent serovar in infants and toddlers (Cui et al., 2009).

Antibiotic resistance to *Salmonella* is one of the most important public health problems worldwide and has increased significantly over recent years due to the long-term use of antibiotics in animal production practices (Barza, 2002). Multidrug-resistant (MDR) *Salmonella* may pose a serious threat to humans through the food chain, potentially contributing to long-term illness, disability, and death (Valdezate et al., 2005). The U.S. Centers for Disease Control and Prevention (CDC) estimated that at least 2 million people in the United States are infected with drug-resistant bacteria each year, resulting in at least 23,000 deaths and posing a serious threat to human health (Hu et al., 2020). *Salmonella* antibiotic resistance has increased over the last 20 years (Zhang et al., 2006). This phenomenon is especially severe in China. Here, *Salmonella* isolates in the 1960s were not multi-drug resistant, but since the mid-1970s, when antibiotics in animal feed became popular, a large number of new drug-resistant strains have emerged (Lin et al., 2004).

According to the *Salmonella* multilocus sequence typing (MLST) database (Maiden et al., 2013), there are more than 20 different sequence types associated with *S.* Derby, and the prevalence of these varies among countries; for example, six different sequence types (ST39, ST40, ST71, ST678, ST682, and ST683) are associated with *S.* Derby in Denmark (Litrup et al., 2010), and there are five in Germany (ST39, ST40, ST71, ST682, and ST774), of which ST39 is the most prevalent (Hauser et al., 2011). In China, there are two main sequence types, ST40 and ST71, with ST40 being the most common (Li et al., 2016), ST40 is also the current most common ST in Shenzhen. ST classification is based on the number of different alleles present, but these different STs are not sufficient to describe the evolutionary relationship of affinities between different isolates. In recent years, rapid developments in technology have meant whole-genome sequencing (WGS) has become more convenient and versatile. In addition to predicting drug resistance genes, WGS data can also be used in species identification, serotype prediction, the screening of virulence genes, and the rapid tracing of disease outbreaks. WGS is gradually becoming the most important prevention and control tool for providing early warnings of infectious disease epidemics (You et al., 2022).

*Salmonella* infection is a major food safety concern, with *S.* Derby rated among the top 10 human-derived *Salmonella* serotypes according to data from the Food Safety Risk Surveillance of Shenzhen (FSS), infectious diarrhea pathogen spectrum sentinel surveillance (IDDS), and food poisoning outbreak surveillance (FDOS) in Shenzhen (Lin et al., 2019). Very little research on the genomics of *S*. Derby has been conducted in China to date, and often the source of illness remains unknown. We sequenced whole genomes of *S.* Derby isolates collected by IDDS, FDOS, and FSS in Shenzhen from 2011 to 2021 and compared them with those of representative global *S.* Derby isolates. Understanding changes in population structure, host associations, resistance characteristics, and the transmission risk of ST40 *S*. Derby in foods in Shenzhen can provide an important reference for subsequent salmonellosis preventive measures and infection source tracing, and the identification of high-risk foods.

## Materials and methods

### Strain sources

The Shenzhen CDC has established a functioning Foodborne Diseases surveillance network consisting of three systems: FDOS, IIDDS, and FSS. We sequenced all the *S*. Derby isolates archived between 2011 and 2021. No statistical methods were used to determine sample size, and there were no data excluded from the analyses. The experiments were not randomized (Yang et al., 2022). FSS isolated 129 *Salmonella* strains from food samples (mainly livestock meat and poultry meat). A sentinel surveillance by IDDS and FDOS collected 188 human samples from stool samples of outpatients with diarrhea. Because data collection is part of the infectious disease surveillance, individual informed consent was waived. All strains were isolated, purified, and cultured using a VITEK2- compact fully automatic microbial identifier (BioMérieux, France) and were identified as *S*. Derby serotype by glass-slide agglutination according to the White Kauffmann-Le Minor method (Grimont and Weill, 2007).

In total, 129 isolates were collected from food, including livestock meat (73.6%, 95/129), poultry meat (13.2%, 17/129), frozen food (4.7%, 6/129), aquatic products (3.9%, 5/129), ready-to-eat foods (3.1%, 4/129), and pastries (1.6%, 2/129). *Salmonella*-infected livestock meat mainly included pig liver (44.2%, 42/95) and pork (42.1%, 40/95), and the others were beef (7.4%, 7/95) and pig kidney (6.3%, 6/95; Supplementary Figure S1A). FSS could not continuously conduct sampling because of new coronavirus epidemic, resulting in no strains isolated in 2020, and only one strain isolated in 2021. However, human-derived strains were detected every year, these data collections are part of infectious disease surveillance and sample collection was less affected by new coronavirus outbreak (Supplementary Figure S1B).

### WGS and genomic datasets

Genomic DNA was extracted using the QIAamp DNA Mini Kit (QIAGEN, Hilden, Germany), according to the manufacturer’s instructions. WGS was performed by Tianjin Novozyme Bioinformatics (Tianjin, China). After passing the quality control assessment, a library was prepared using the NEBNextUltra DNA Library Prep Kit for Illumina (NEB, United States) with an average insert size of 350 bp for sequencing on the Illumina NovaSeq 6000 platform. The average read length was 150 bp, the minimum theoretical coverage was 100×, and an average of 1.2 Gb clean data were produced for each isolate. The data presented in the study are deposited in the National Center for Biotechnology Information (NCBI) sequence read archive (SRA) under BioProject: PRJNA883032.

A total of 390 genomes were analyzed in this study, including 314 newly sequenced *S.* Derby genomes from Shenzhen isolates and 76 *S.* Derby ST40 genomes from 12 countries worldwide obtained from the EnteroBase^1^ (Zhou et al., 2020). Single SRA accession numbers of all the strains and associated epidemiological data are listed in Supplementary Table S1.

### Bioinformatics analysis

Genomic contig sequences were obtained by *de novo* sequence splicing of genomic data from each strain using Shovill (v. 1.0.4; Seemann, 2018). Raw data were subjected to quality control using Trimmomatic^2^ (v. 0.39; Bolger et al., 2014) to obtain valid data. Genome assembly quality was assessed using QUAST^3^ (Gurevich et al., 2013), and the mean N50 was 313,894 bp. *Ab initio* genome assembly was performed using SPAdes^4^ gene assembly software (v. 3.9.1; Bankevich et al., 2012). Strain 2014LSAL02547 (NCBI no. CP029486) was used as a reference strain for ST40 analysis (Sevellec et al., 2018). Mapping-based single-nucleotide polymorphism (SNP) typing was performed using Snippy^5^ (v. 4.3.6; Olawoye et al., 2020). Gubbins^6^ (Croucher et al., 2015) with default parameters was used for core genome de-recombination. The resulting SNP matrix of preserved sites was then used to construct a phylogeny tree with FastTree (v. 2.1.10; Price et al., 2009) software and the maximum likelihood method, which was embellished using ITOL^7^ (Letunic and Bork, 2021).

### Analysis of antibiotic resistance genes, *Salmonella* pathogenicity islands, plasmid replicons, and multi-locus sequence typing

Resistance genes and chromosomal mutation regions in assembled contigs were identified using Resfinder (v 0.3.2; Zankari et al., 2012) with an identity threshold of 75% and coverage of 75%. *Salmonella* pathogenicity islands (SPIs) were detected using SPIfinder (v. 1.0; Roer et al., 2016) with default settings of coverage ≥75% and identity ≥75%. PlasmidFinder (v 0.2.0.1; Carattoli and Hasman, 2020) was applied to predict plasmid replicons with sequence identity ≥80% and coverage ≥80%.

Each MLST sequence type (MLST-ST) was obtained by scanning the sequences of seven house-keeping genes (a*roC*, *dnaN*, *hemD*, *hisD*, *purE*, *sucA*, and *thrA*) against PubMLST typing schemes using mlst.2 (Carattoli and Hasman, 2020).

### Antibacterial drug susceptibility testing

The Gram-negative aerobic bacterial susceptibility panel (Shanghai Xingbai Biotechnology Co., Ltd.) was used according to the recommendations of Clinical and Laboratory Standards Institute document M100-S30, using *Escherichia coli* ATCC25922 as the quality control strain. The minimum inhibitory concentrations of 17 antibacterial drugs, ampicillin (AMP), ampicillin/sulbactam (AMS), tetracycline (TET), meropenem (MEM), polymyxin E (CT), ertapenem (ETP), ceftazidime/avibactam (CZA), tigecycline (TGC), cefotaxime (CTX), ceftazidime (CAZ), ciprofloxacin (CIP), azithromycin (AZI), chloramphenicol (CHL), nalidixic acid (NAL), streptomycin (STR), trimethoprim/sulfamethoxazole (SXT), and amikacin (AMK), were tested for *S.* Derby strains.

## Results

### Sequence type

Two different ST profiles were identified among the 317 studied genomes: ST40 [*aroC* (19), *dnaN* (20), *hemD* (3), *hisD* (20), *purE* (5), *sucA* (22), *thrA* (22)] and ST71 [*aroC* (39), *dnaN* (35), *hemD* (8), *hisD* (36), *purE* (29), *sucA* (9), and *thrA* (36)]. The most frequent profile in the collection was ST40 (*n* = 314 genomes), followed by ST71 (*n* = 3). All ST40 isolates were included in this study.

### Phylogenetic analysis of *Salmonella* Derby

**Figure 1:**
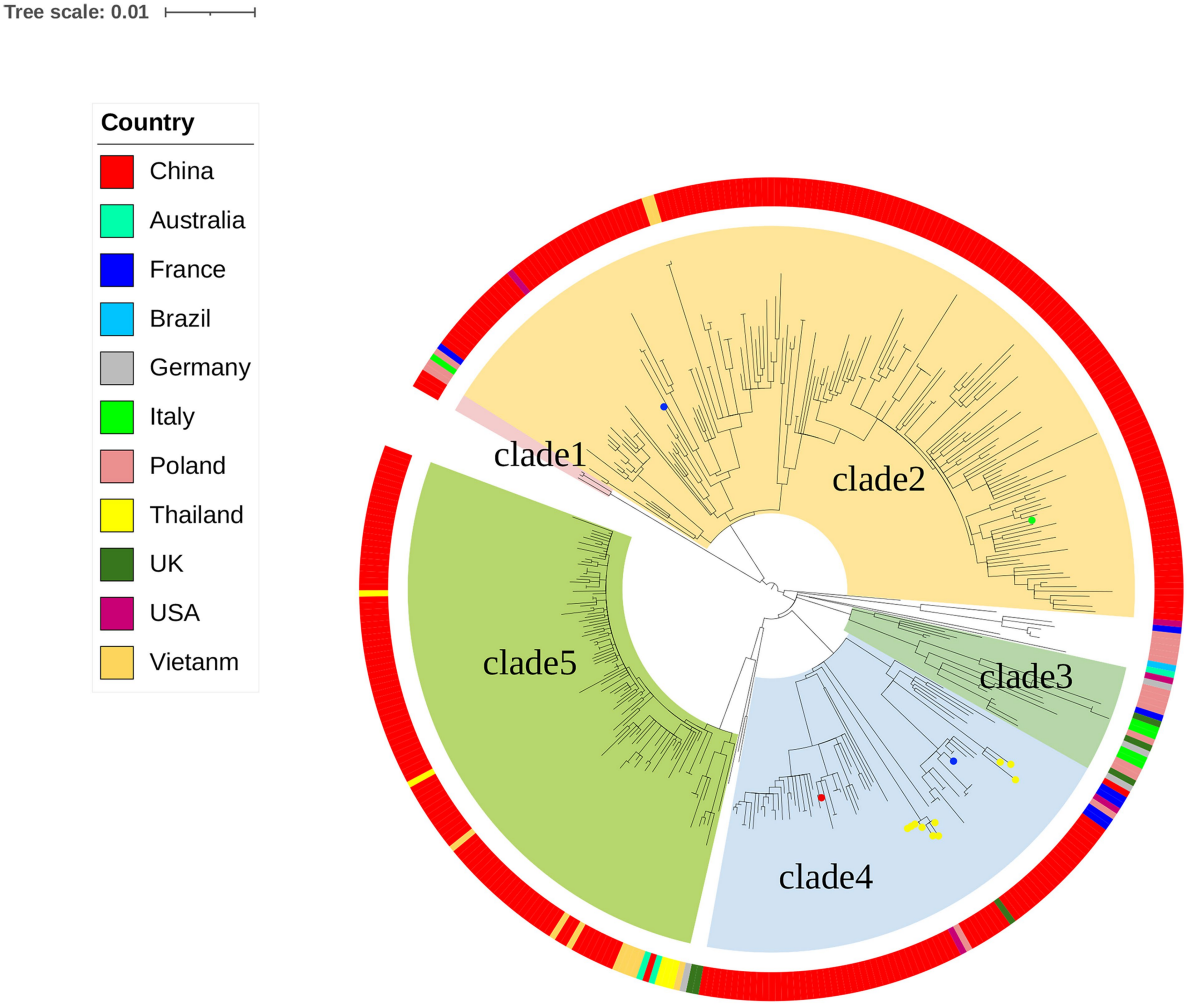
Maximum likelihood phylogenetic tree of 390 *Salmonella* strains. The background colors of the phylogenetic branches indicate the different evolutionary branches. Outer circles indicate different countries and the circles at the ends of the branches indicate other cities in China (blue, Shandong, China; green, Yangzhou, China; yellow, Taiwan, China; red, Tibet, China).

**Figure 2:**
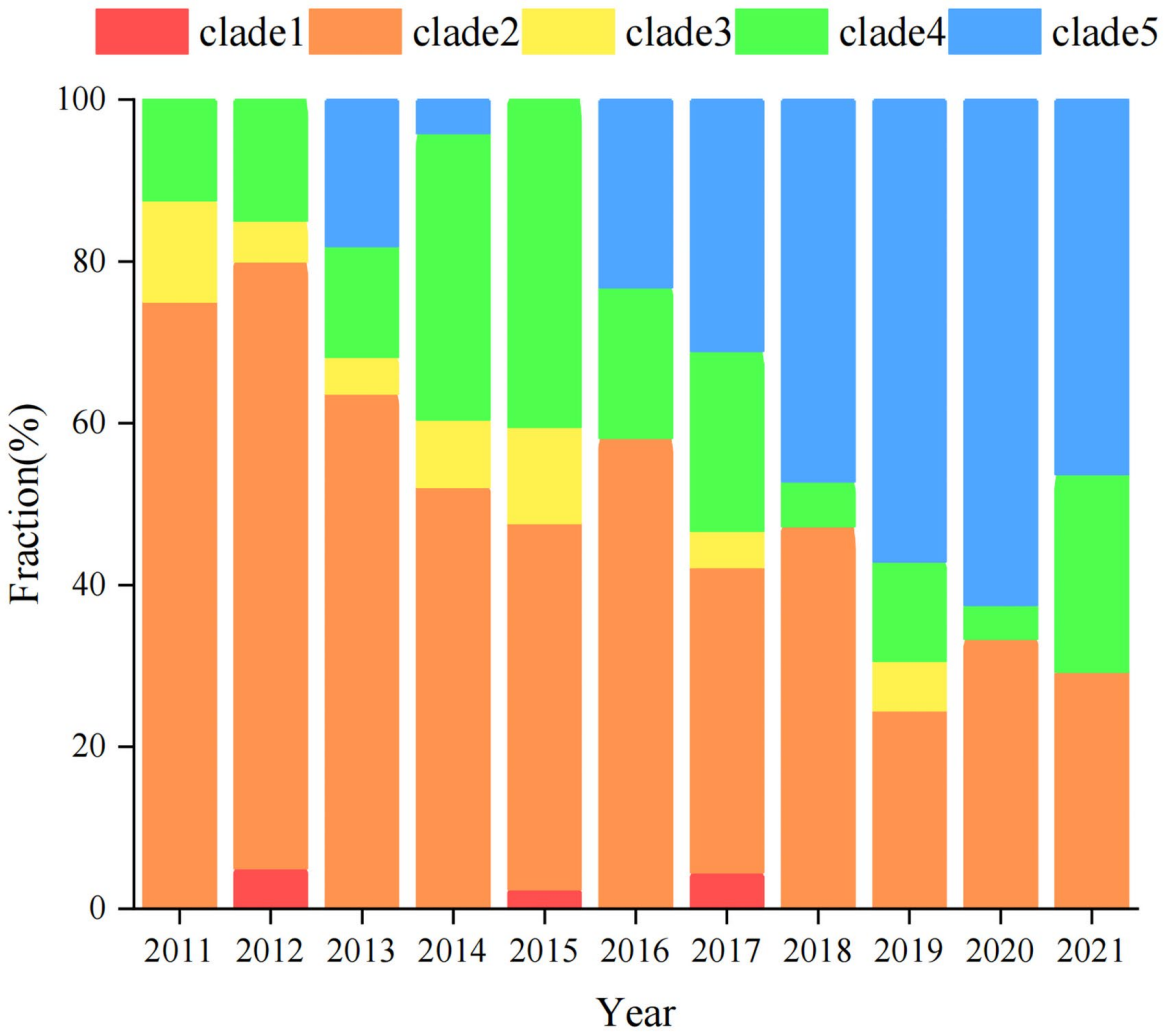
Distribution of *S.* Derby ST40 evolutionary branches globally. Different strip colors indicate different clades, clade1 (red), clade2 (orange), clade3 (yellow), clade4 (green), and clade5 (blue).

The genomes of local *S.* Derby ST40 samples from Shenzhen (*n* = 314) and 76 globally prevalent representative strains from France (*n* = 6), Germany (*n* = 4), Italy (*n* = 5), United Kingdom (*n* = 6), Poland (*n* = 17), Vietnam (*n* = 10), Thailand (*n* = 5), United States (*n* = 5), Australia (*n* = 3), Brazil (*n* = 1), and other Chinese provinces (*n* = 14) were analyzed based on SNP frequencies. A total of 4,963 core-SNP loci were detected. Chinese isolates were found to have significant diversity and could be divided into five main and several small branches, with distances between strains within each branch of <215 SNPs. Chinese isolates were mainly concentrated in Clades 2 (41.3%, 161/390), 5 (24%, 94/390), and 4 (17.9%, 70/390), with the remaining isolates concentrated in Clade 1 (0.8%, 3/390). Strains from Shenzhen, part of the Chinese inland city of Shandong, and other Asian countries (Thailand and Vietnam) were closely related. Strains from European countries were concentrated in an independent branch of Clade 3 and were distantly related to the Chinese strains (Figure 1). During the survey, Clades 2, 4, and 5 were dominant. Clade 5 appeared in 2013 and stabilized after 2016; both Clades 2 and 4 persisted during the 11 sampled years, but the proportion of the latter was variable, with multiple co-existing clonal clusters appearing in 2017 (Figure 2).

**Figure 3:**
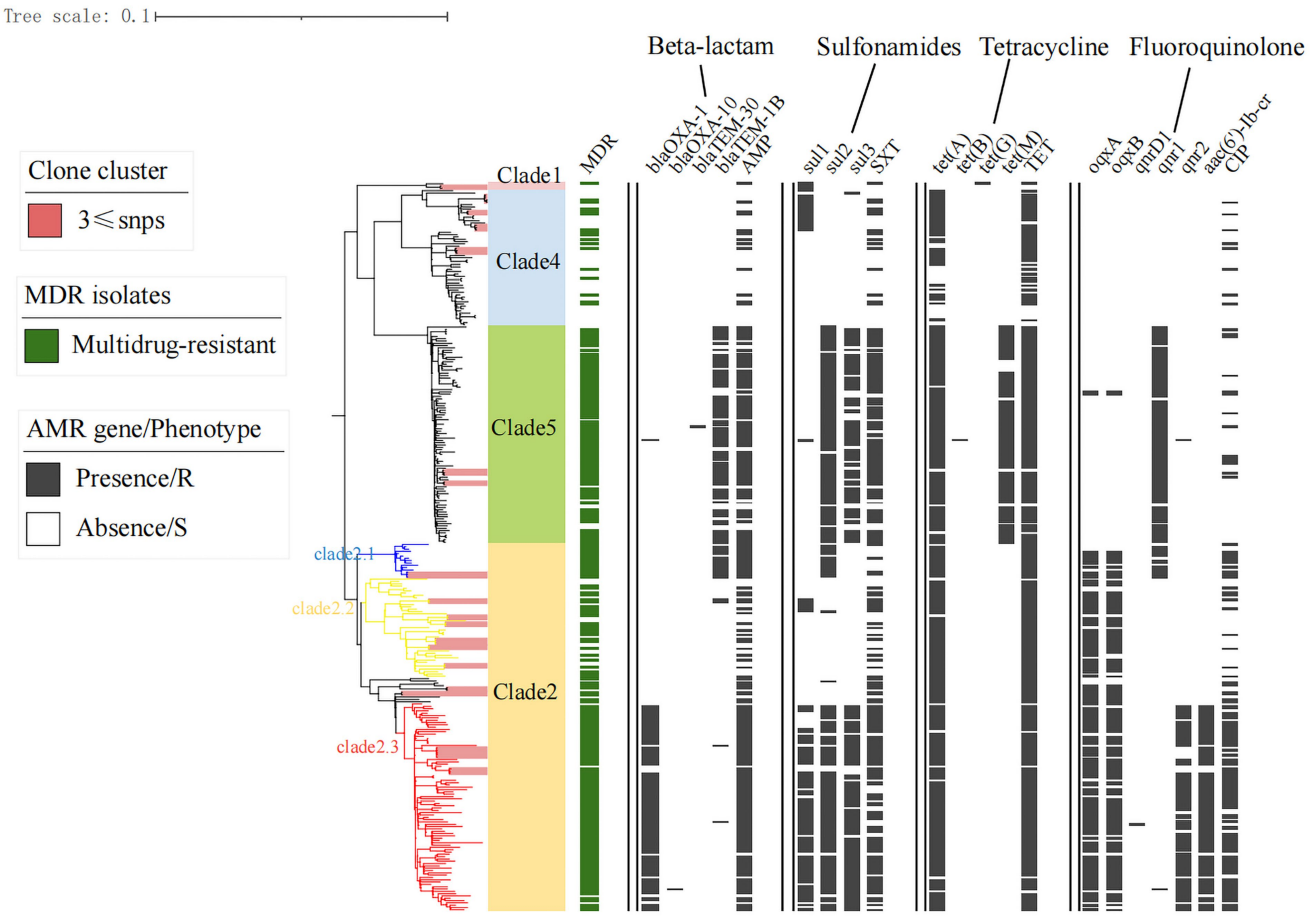
Antimicrobial resistance and gene distribution of Shenzhen strains. The maximum likelihood tree of 314 Shenzhen isolates is shown on the left, with the evolutionary branch of ST40 *S*. Derby in background color (as in Figure 1); on the right, the distribution of multidrug-resistant (MDR) isolates (green bars), representative isolates for phenotypic testing, and the presence (black bars) or absence (white bars) of antimicrobial resistance-associated mutations and genes.

**Figure 4:**
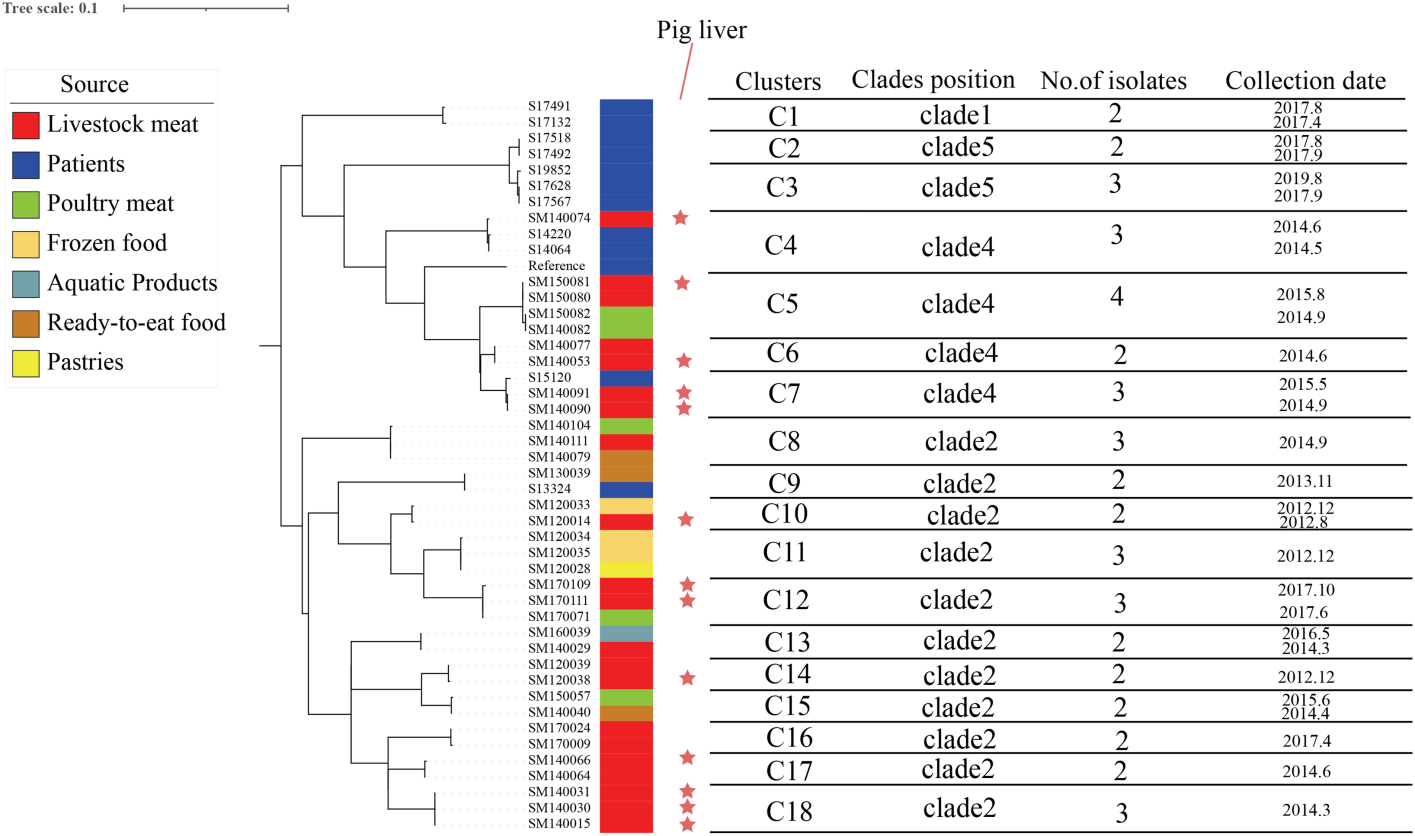
Maximum likelihood phylogenetic tree and strain information for 18 clonal clusters. The maximum likelihood tree of 18 clonal clusters is shown on the left; the color of the strip reports the origin of the strain: patients (blue), livestock meat (red), poultry meat (green), frozen food (earthy yellow), pastries (bright yellow), ready-to-eat food (brown), and aquatic products (lake blue); the red stars indicate that the strains were isolated from pig liver; and the contents of the line table contain the clades position, no. of isolates, and collection date.

A total of 5,408 core-SNP sites were detected in the 314 strains isolated from Shenzhen. With reference to the cutoff set for *S. enteritidis* outbreak clonal clusters, we identified clonal clusters in the Shenzhen *S*. Derby ST40 maximum likelihood evolutionary tree, in which there were three SNPs across strains (Taylor et al., 2015; Jiang et al., 2020). There were 11, 4, and 2 clusters in Clades 2, 4, and 5, respectively (Figure 3). In an evolutionary tree constructed using ≤3 SNPs between two strains, 18 clusters involved 45 strains, with 2–4 isolates per cluster, differing by 0 ~ 3 SNPs; three clonal clusters included only human strains (C1, C2, and C3), three clusters had both human and food strains (C4, C7, and C9), and 12 clusters comprised only food-derived strains (C5, C6, C8, C10, C11, C12, C13, C14, C15, C16, C17, and C18; Figure 4). Among the 45 strains involved, 34 were food-derived and 11 were human-derived. Most of the 34 food-derived strains (13/34), and all food-derived strains on C4 and C7, were from pig liver. We also found that most of the strains in the same clusters came from the same market or supermarket and were isolated at similar times (Figure 4).

### Drug resistance genes, plasmid replicons, and virulence gene assays

To characterize the AMR profile of Shenzhen *S.* Derby isolates, we first screened the genome sequence data to identify AMR-associated mutations and genes. A total of 45 different AMR mutations/genes were detected in 11 classes, including those involved in resistance to aminoglycosides (13 genes), β-lactams (7 genes), sulfonamides (3 genes), TET (4 genes), fluoroquinolones (3 mutations and 6 genes), CHL (4 genes), methicillin (3 genes), macrolides (1 gene), polymyxins (1 gene), fosfomycin (1 gene), and rifampicin (2 genes). Aminoglycoside *aac(6′)-Iaa* and fosfomycin *fosA7*-resistance genes were detected in all strains. The quinolone-resistance genes mainly comprised *oqxA/B* (42%), *aac(6′)-Ib-cr* (25.6%), and *qnrD1/S1/S2* (55.5%). The plasmid gene *qnr* was dominated by *qnrS1*. One and eight strains were detected carrying the *gyrA* p.S83F and p.D87N mutations, respectively. The *bla*_TEM-1B_ allele was the most common β-lactam-resistance gene (28.7%), followed by *bla*_OXA-1_ (25.9%), which was also plasmid-mediated. Sulfonamide-, chloramphenicol-, and rifampicin-resistance genes were dominated by *sul2* (58.4%), *floR* (53%), and *arr-3* (25.2%), respectively.

Fifty-eight strains carrying 17 incompatibility group (Inc) plasmid replicons were detected among the 314 *S.* Derby ST40 strains; the three most common were IncI1_1 (8.28%, 26/314), IncHI2_1 (7.32%, 23/314), and IncHI2A_1 (7.00%, 22/314), with each of the 58 strains carrying 1–5 plasmid replicons. All strains carried six pathogenicity islands (SPI-1, SPI-2, SPI-3, SPI-4, SPI-5, and SPI-9) simultaneously, and one strain carried both SPI-13 and SPI-14 (Table 1).

**Table 1:** Results of plasmid replicon and virulence gene analysis of *Salmonella* Derby ST40 strains (*n* = 314).

*S.* Derby clade	No. of isolates	MDR	Plasmid replicons	SPIs
Clade 1	3	33%	-	SPI-1 (*n* = 3); SPI-2 (*n* = 3); SPI-3 (*n* = 3); SPI-4 (*n* = 3); SPI-5 (*n* = 3); SPI-9 (*n* = 3)
Clade 2	159	84.9%	IncHI2A_1 (*n* = 16); IncHI2_1 (*n* = 16); IncI1_1 (*n* = 11); IncX1_4 (*n* = 6); IncFIA_1 (*n* = 1); IncFII-1 (*n* = 3); IncL/M (pOXA-48)_1 (*n* = 1); IncQ1_1 (*n* = 11); IncX3_1 (*n* = 1); IncX4_1 (*n* = 1)	SPI-1 (*n* = 159); SPI-2 (*n* = 159); SPI-3 (*n* = 159); SPI-4 (*n* = 159); SPI-5 (*n* = 159); SPI-9 (*n* = 159); SPI-13 (*n* = 1); SPI-14 (*n* = 1)
Clade 2.1	15	100%	IncI1_1 (*n* = 2); IncFIA_1 (*n* = 1); IncFII-1 (*n* = 2); IncHI2_1 (*n* = 9); IncHI2A_1 (*n* = 9); IncQ1_1 (*n* = 7)	SPI-1 (*n* = 15); SPI-2 (*n* = 15); SPI-3 (*n* = 15); SPI-4 (*n* = 15); SPI-5 (*n* = 15); SPI-9 (*n* = 15)
Clade 2.2	43	60.5%	IncX1_4 (*n* = 6); IncHI2A_1 (*n* = 6); IncHI2_1 (*n* = 6); IncQ1_1 (*n* = 1); IncI1_1 (*n* = 3)	SPI-1 (*n* = 43); SPI-2 (*n* = 43); SPI-3 (*n* = 43); SPI-4 (*n* = 43); SPI-5 (*n* = 43); SPI-9 (*n* = 43)
Clade 2.3	90	95.6%	IncI1_1 (*n* = 6); IncHI2_1 (*n* = 1); IncFII-1 (*n* = 1); IncL/M (pOXA-48)_1 (*n* = 1); IncX3_1 (*n* = 1); IncX4_1 (*n* = 1); IncQ1_1 (*n* = 7)	SPI-1 (*n* = 90); SPI-2 (*n* = 90); SPI-3 (*n* = 90); SPI-4 (*n* = 90); SPI-5 (*n* = 90); SPI-9 (*n* = 90)
Clade 4	58	27.6%	IncHI2A_1 (*n* = 4); IncHI2_1 (*n* = 4); IncQ1_1 (*n* = 4); IncB/O/K/Z_1 (*n* = 1); IncI1_1 (*n* = 4)	SPI-1 (*n* = 58); SPI-2 (*n* = 58); SPI-3 (*n* = 58); SPI-4 (*n* = 58); SPI-5 (*n* = 58); SPI-9 (*n* = 58)
Clade 5	94	89.4%	IncI1_1 (*n* = 9); IncFIA_1 (*n* = 1); IncHI1A_1 (*n* = 1); IncX1_1 (*n* = 2); IncQ1_1 (*n* = 2); IncHI2A_1 (*n* = 1); IncHI2_1 (*n* = 1)	SPI-1 (*n* = 94); SPI-2 (*n* = 94); SPI-3 (*n* = 94); SPI-4 (*n* = 94); SPI-5 (*n* = 94); SPI-9 (*n* = 94)

MDR, multidrug resistance; SPI, *Salmonella* pathogenicity island.

### Antimicrobial susceptibility

The resistance genes found in *Salmonella* in food and patient samples from the 314 strains isolated in Shenzhen were generally concordant with phenotype testing results. The 314 strains of *S.* Derby ST40 were resistant to 16 antibiotics, not including TGC, to varying degrees (0.3–90.54%; Table 2). TET resistance was closely related to the presence of *tet(A)*, with 274 (95.5%) of 287 TET-resistant strains encoding *tet(A)*; while resistance to TGC, another antibiotic in the tetracycline class, was not detected. Resistance to β-lactams, including AMP (68.5%, 215/314), AMS (14%, 44/314), CZA (0.3%, 1/314), CAZ (1.3%, 4/314), and CTX (1.6%, 5/314), was detected, of which 79.5% (171/215) encoded at least one type of β-lactamase-associated gene, with *bla*_OXA-1_ (38.1%, 82/215) and *bla*_TEM-1B_ (42.3%, 91/215) being the most common. In addition, resistance to the sulfonamide antibiotics SXT (62.4%, 196/314) and CHL (75.4%, 237/314) was detected; 83.2% (163/196) of strains resistant to sulfonamide antibiotics encoded at least one sulfonamide-resistance gene, and 73.4% of chloramphenicol-resistant strains contained *floR* (70.5%, 167/237) and *cmlA1* (60.8%, 144/237) genes. Of the two azithromycin-resistant strains, one carried the *mph(A)* resistance gene. Six strains were resistant to the quinolone antibiotic NAL (42%, 132/314) and carried the *gyrA* p.S83F or p.D87N mutations, while four CIP-resistant strains (40%, 126/314) carried the *gyrA* p.D87N mutation. Resistance to CTX, AMK, CZA, ertapenem, and meropenem was detected in one strain each. By comparison, we found that both food- and human-derived strains were severely resistant to AMP, TET, and AZI, while food-derived strains were more resistant to CTX, CZA, and CHL than human-derived strains (Supplementary Table S2).

**Table 2:** Drug-resistant phenotypes of *S.* Derby ST40 strains (*n* = 314).

Antibacterial drug types	Antimicrobial agent	R		I		S	
		No. of isolates	Rate (%)	No. of isolates	Rate (%)	No. of isolates	Rate (%)
Penicillin	AMP	215	68.5	2	0.63	97	30.9
	AMS	44	14	161	51.3	109	34.7
Tetracycline	TET	287	90.54	0	0	27	8.6
	TGC	0	0	0	0	314	100
Polymyxins	CT	1	0.32	313	99.68	0	0
Carbapenems	ETP	1	0.32	0	0	313	99.68
	MEM	1	0.32	0	0	313	99.68
Cephalosporins	CZA	1	0.32	0	0	313	99.68
	CTX	5	1.6	0	0	309	98.4
	CAZ	4	1.3	0	0	313	99.7
Quinolones	CIP	122	38.9	16	5.1	176	56.1
	NAL	132	42	0	0	182	58
Aminoglycosides	AMK	1	0.32	0	0	313	99.7
	STR	107	34.1	0	0	207	65.9
Macrolides	AZI	2	0.63	0	0	312	99.36
Amido alcohols	CHL	237	75.4	0	6.31	57	18.2
Sulfonamides	SXT	196	62.4	0	0	118	37.6

R, antibiotic resistance; I, antibiotic intermediary; S, antibiotic sensitivity; AMP, ampicillin; AMS, ampicillin-sulbactam; AZI, azithromycin; AMK, Amikacin; CT, Polymyxin E; CZA, ceftazidime/avibactam; CTX, cefotaxime; CAZ, Ceftazidime; CIP, ciprofloxacin; CHL, chloramphenicol; ETP, ertapenem; MEM, meropenem; NAL, nalidixic acid; STR, Streptomycin; SXT, trimethoprim/sulfamethoxazole; TET, tetracycline; TGC, tigecycline; Breakpoints (in milligrams per liter) are as follows: AMP, S ≤ 8, I = 16, and R ≥ 32; AMS, S ≤ 8, I = 16, and R ≥ 32; AZI, S ≤ 16 and R ≥ 32; AMK, S ≤ 16, I = 32, and R ≥ 64; CT, S = 2, I ≤ 4, and R ≥ 8; CZA, S ≤ 2 and R ≥ 4; CTX, S ≤ 1, I = 2, and R ≥ 4; CAZ, S ≤ 4, I = 8, and R ≥ 16; CIP, S ≤ 1, I = 2, and R ≥ 4; CHL, S ≤ 8, I = 16, and R ≥ 32; ETP, S ≤ 0.5, I = 1, and R ≥ 2; MEM, S ≤ 1, I = 2, and R ≥ 4; NAL, S ≤ 16 and R ≥ 32; STR, S ≤ 8, I = 16, and R ≥ 32; SXT, S ≤ 2/38 and R ≥ 4/76; TET, S ≤ 4, I = 8, and R ≥ 16; TGC, S ≤ 2, I = 4, and R ≥ 8.

Notably, unlike the plasmids and virulence factors, the drug-resistance genes/phenotypes were associated with evolutionary branches to some extent. There were higher rates of β-lactam, sulfonamide, tetracycline, and quinolone-related resistance genes/phenotypes in Clade 2.1, 2.3, and 5 strains than those of other evolutionary branches (Figure 3). MDR strains were defined as those resistant to three or more antibiotics; 6% (18/314) of strains were susceptible to all 17 antimicrobial drugs, and almost 75% (236/314) exhibited MDR, with the most common MDR pattern being AMP-TET-CIP-CHL-NAL-STR-SXT (Supplementary Table S3).

## Discussion

Our WGS-based reconstruction of the population structure of Shenzhen *S.* Derby ST40 isolates in the context of the global epidemic revealed the genomic diversity of the bacteria and the associations among various hosts in the Shenzhen region. This is the first study of the genomic epidemiology and drug resistance characteristics of *S.* Derby ST40 in China, and it was conducted over an extended period of time. Here, we traced the high-risk food and pig liver, and identified two evolutionary branches. Some of those strains clustered closely together indicating a potential foodborne outbreak with pig liver as vehicle. Isolates from Shenzhen showed simultaneous susceptibility to cephalosporins while being severely resistant to TET, CHL, and AMP. *S.* Derby is a significant serotype, but little is known about its genetic diversity in China. Our data expand on the publicly available sequence data and information on the genetic diversity of *S*. Derby in Shenzhen. At the same time, this study had some limitations. Because of the impact of the new coronavirus epidemic, FSS sampling was not continuous, resulting in a discontinuous collection of food-derived strains in the last 2 years.

Shenzhen is a developed metropolis in the south of China with a population of over 20 million. It has little arable land and no farms for raising poultry or livestock; therefore, the majority of food is imported from other Chinese cities and other countries. According to the global phylogenetic evolutionary tree constructed in this study, Shenzhen *S.* Derby strains are closely related to those from the inland Chinese city of Shandong as well as other Asian nations, including Thailand and Vietnam, while strains in European nations are primarily concentrated in an independent branch of Clade 3 and are more distantly related to Chinese strains. However, fewer prevalent representative strains from other regions have been uploaded to the public database, and more representative strains need to be collected to construct a global evolutionary tree for a more comprehensive picture of their prevalence. The ubiquity of *S.* Derby ST40 in Shenzhen for 11 years has resulted in significant diversity, and strains in all four evolutionary branches were present, indicating that the serotype has undergone microevolution over the 11-year epidemic. In recent years, outbreaks in the area have been dominated by Clades 2, 4, and 5, which may be connected to their higher levels of adaptability and MDR. The MDR rate of Clade 2 (especially Clades 2.1 and 2.3) and Clade 5 was 80% or more (Table 1).

Pigs have the potential to spread *Salmonella* infection during group feeding. *S.* Derby can also be found in the area where the pig carcasses are divided. According to U.S. CDC data, *S.* Derby is most frequently isolated from pig production units. *S.* Derby can cause long-term infections in pigs and can remain in several organs for a long time, which explains why *Salmonella* is easily isolated from pig organ parts, and this leads to contaminated pork at slaughter (Cevallos-Almeida et al., 2019). In our study, we identified 18 clonal clusters based on a threshold of three SNPs for the definition of *S. enteritidis* outbreaks (Taylor et al., 2015; Jiang et al., 2020), and 15 of the 34 strains in food were isolated from pig liver. Three clonal clusters (C4, C7, and C9) were detected in both patients and food, and the food-derived strains of two of the clonal clusters (C4 and C7) were isolated from pig liver and were in Clade 4 (Figure 4). We surmised that pig liver poses a high risk for foodborne illness outbreaks. We also discovered that the majority of foods containing bacteria in the same clonal clusters originated from the same market or supermarket and that there were various food types with comparable collection times in the same clusters. Furthermore, our market research found that different meats are sold on the same counter at supermarkets or markets and that the same cutting board and knives are used for most meats, indicating that there may be cross-contamination during food processing and selling. Our results suggest that WGS and clonal cluster analysis can be used for the identification of high-risk food types, pointing to new avenues for subsequent outbreak prevention and control strategies. These actions are also consistent with a positive One Health vision: pathogens supplied by various stakeholders from many sources (including human clinical samples, animal, food, and environmental samples) can be pooled and studied for various purposes across various analytical platforms (Timme et al., 2020), which provides a large amount of data that can be used by public health agencies for outbreak detection and tracking (Marc et al., 2016).

Plasmids are circular DNA molecules that can replicate independently from the bacterial chromosome and carry genetic material such as virulence and drug-resistance genes. Through horizontal gene transfer, mobile plasmids can spread drug resistance and virulence rapidly among bacteria of the same or other species, increasing the challenge of treating clinical infectious illnesses (Liu et al., 2009). The propagation of β-lactamase- and quinolone-resistance genes is directly related to the detection of both IncI1- and IncHI2-type plasmids (Sukmawinata et al., 2020). SPIs are located on chromosomes and code virulence-associated proteins that help *Salmonella* to invade, reproduce, and spread within its complex environment propagation. There are 23 known SPIs, of which SPI-1 to SPI-5 are shared by all *S. enterica* serovars, while the others are scattered among other serotypes. In our analysis, all strains had six pathogenicity islands (SPI1 to SPI5 and SPI9), while SPI13 and SPI14 were recognized in only one strain. SPI-9 is strongly related to biofilm development (Latasa et al., 2005), as it encodes proteins that share sequence similarity with members of the Bap family. In *Staphylococcus aureus*, Bap is a cell wall protein that strongly encourages biofilm development and has the potential to stimulate the emergence of drug-resistant strains (Cucarella et al., 2001). Prior research found SPI-13 and SPI-14 only in *S. typhimurium* and *S. enteritidis* (Shah et al., 2005), and the current study was the first to identify these islands in *S.* Derby. However, their mechanisms of action are still unclear and need further study.

Clarification of the antibiotic susceptibility profile of *S.* Derby will inform researchers on how to develop more effective clinical treatments. Unsurprisingly, strains from food sources had higher levels of resistance to antibiotics, such as TET, AMP, CHL, sulfonamides, and NAL, than those from human sources, as these drugs are frequently included in animal feed to treat illness or boost growth. Because of worries over the emergence of antibiotic resistance and the transmission of antibiotic-resistance genes from animals to people, EU countries began to outlaw the use of antibiotics as growth promoters in 2006 (Castanon, 2007). Recently, numerous other nations have documented significant *S.* Derby drug resistances to these widely used medications. In Sichuan and Guangzhou, China, TET is frequently used in feed for poultry livestock, and the food-derived strains identified in this study were primarily isolated from livestock meat. Because people in China consume a lot of pork, there is a chance that the increasing antibiotic resistance in farmed animals will spread to humans through the food chain and result in the failure of clinical antibiotics. NAL is a first-generation quinolone that bacteria have quickly developed resistance to. We discovered that the rate of NAL resistance of bacteria in food (67.4%) in China was significantly greater than that in Europe (10%; Jong et al., 2009). We concluded that to prevent the further emergence and spread of antibiotic resistance and to ensure food safety in China, intervention measures must be developed to manage food sources and restrict the use of antibiotics in animal husbandry.

Finally, this study reconstructed the WGS-based population structure of *S*. Derby ST40 in Shenzhen in the context of globally endemic representative strains. The kinship evolutionary tree formed five evolutionary branches, revealing that *S*. Derby ST40 strains in this region have closer affinities with endemic strains in other Asian countries than those in European countries. By constructing an evolutionary tree of relatedness, we discovered multiple epidemic clonal turnovers in the region over the 11 years of sampling. Notably, this study identified three currently prevalent evolutionary branches with high-resistance and high-transmission risk, Clades 2, 4, and 5, and we identified a high-risk food, pig liver. This information has important implications for salmonellosis prevention, source tracing, and risk-factor analysis, as well as laying the groundwork for future *S*. Derby studies.

## Data availability statement

The datasets presented in this study can be found in online repositories. The names of the repository/repositories and accession number(s) can be found in the article/Supplementary material.

## Author contributions

ML, MJ, and QH conceived and designed the study. YQ, CY, LH, LW, SW, and XS performed article data collection. YS, ML, and LX experiments and data analysis were conducted. ML wrote the original draft. CY, LX, and YJ conducted raw letter analysis guidance. ML, CY, and QH reviewed and revised the paper. All authors contributed to the article and approved the submitted version.

## Funding

This work was supported by the Sanming Project of Medicine in Shenzhen under grant (no. SZSM201811071); Shenzhen Key Medical Discipline Construction Fund (SZXK064); Key scientific and technological project of Shenzhen Science and Technology Innovation Committee (KCXFZ202002011006190); and Non-profit Central Research Institute Fund of Chinese Academy of Medical Sciences (2020-PT330-006).

## Conflict of interest

The authors declare that the research was conducted in the absence of any commercial or financial relationships that could be construed as a potential conflict of interest.

## Publisher’s note

All claims expressed in this article are solely those of the authors and do not necessarily represent those of their affiliated organizations, or those of the publisher, the editors and the reviewers. Any product that may be evaluated in this article, or claim that may be made by its manufacturer, is not guaranteed or endorsed by the publisher.

## Footnotes

^1^ http://enterobase.warwick.ac.uk

^2^ https://github.com/timflutre/trimmomatic

^3^ https://github.com/ablab/quast

^4^ https://github.com/ablab/spades

^5^ https://github.com/ablab/snippy

^6^ https://github.com/nickjcroucher/gubbins

^7^ https://itol.embl.de/
