# Supplementary figures and images for: Population dynamics and antimicrobial resistance of Salmonella Derby ST40 from Shenzhen, China
Source: Front Microbiol. 2022 Dec 20;13:1065672. doi: 10.3389/fmicb.2022.1065672 (PMC9808032; doi:10.3389/fmicb.2022.1065672)

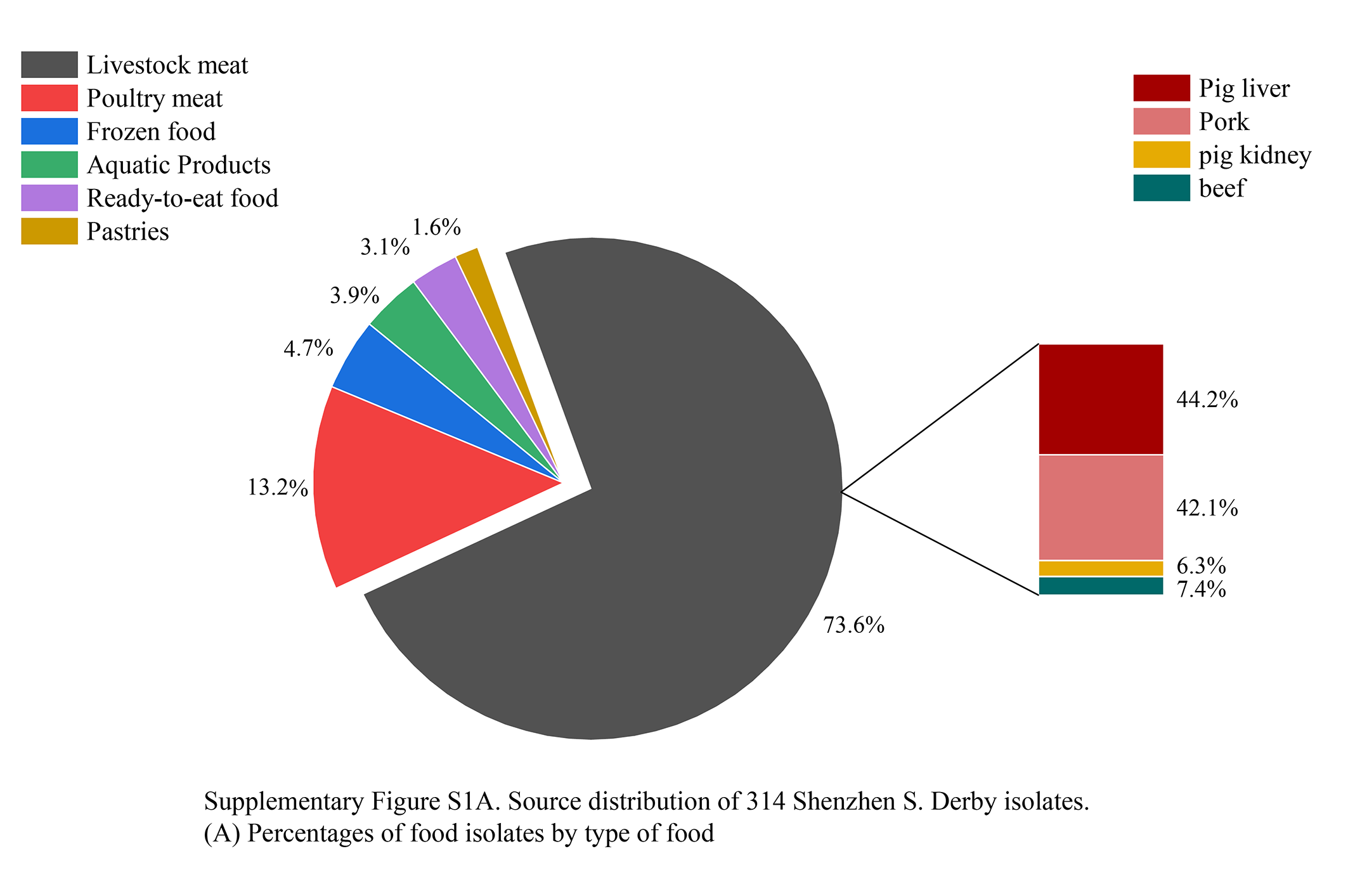

Supplement: Supplementary file 4 [file Image_1.tif]

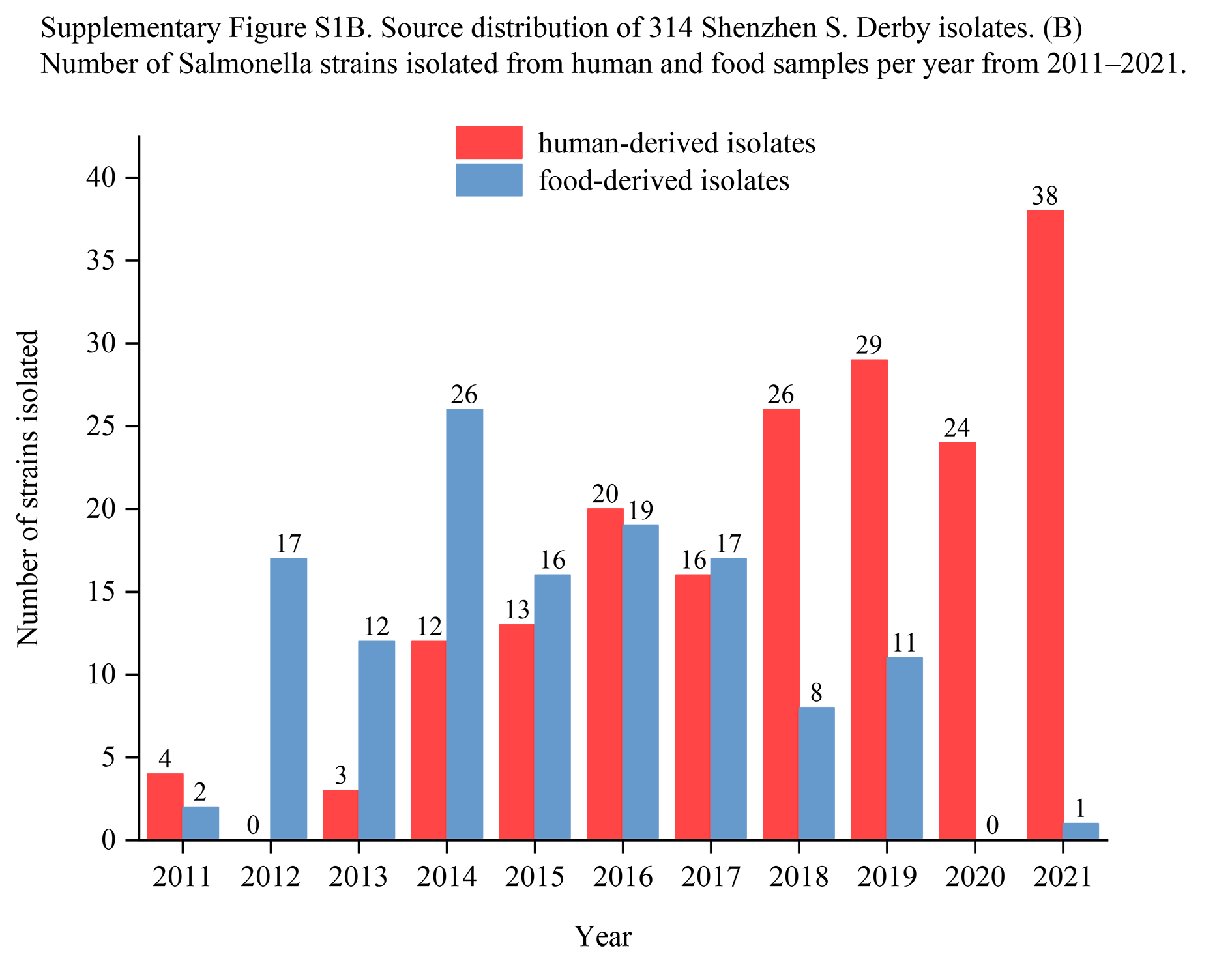

Supplement: Supplementary file 5 [file Image_2.tif]
